# Reviewer acknowledgement 2015

**DOI:** 10.1186/s12977-016-0288-3

**Published:** 2016-08-03

**Authors:** Andrew Lever, Mark A. Wainberg

**Affiliations:** 1 Cambridge, UK; 2 Montreal, Canada

**Contributing reviewers**

Retrovirology would like to sincerely thank the following for giving their time and expertise to review manuscripts for the journal in 2015. Their support for the journal is greatly appreciated.

**Philippe Afonso**

France

**Ramesh Akkina**

USA

**Lorraine Albritton**

USA

**Jose Alcami**

Spain

**Zandrea Ambrose**

USA

**Dong Sung An**

USA

**Nnacie Archin**

USA

**Joop Arends**

Netherlands

**Maria Ariza**

USA

**James Arthos**

USA

**Jan Balzarini**

Belgium

**Charles Bangham**

UK

**Norbert Bannert**

Germany

**Benoit Barbeau**

Canada

**Eric Barklis**

USA

**Jagadeesh Bayry**

France

**Karen Beemon**

USA

**Brendan Bell**

Canada

**Richard Benarous**

France

**Serge Benichou**

France

**Monsef Benkirane**

France

**Yamina Bennasser**

France

**Edward Berger**

USA

**Nicole Bernard**

Canada

**Neil Berry**

UK

**Lionel Berthoux**

Canada

**Paul Bieniasz**

USA

**Kate Bishop**

UK

**Adriano Boasso**

UK

**Eli Boritz**

USA

**Steven Bosinger**

USA

**Alberto Bosque**

USA

**Fadila Bouamr**

USA

**Martine Braibant**

France

**Jason Brenchley**

USA

**Mark Brockman**

Canada

**Zabrina Brumme**

Canada

**Michael Bukrinsky**

USA

**Gregory Burton**

USA

**Vincenzo Calvanese**

USA

**Carol Carter**

USA

**Shan Cen**

China

**Nathalie Chazal**

France

**Benjamin Chen**

USA

**Peter Cherepanov**

UK

**Jakub Chojnacki**

UK

**Tae-Wook Chun**

USA

**Tomas Cihlar**

USA

**Andrea Cimarelli**

France

**Angela Ciuffi**

Switzerland

**Alan Cochrane**

Canada

**Eric Cohen**

Canada

**Julio Coll**

Spain

**Ronald Collman**

USA

**Rafael Contreras Galindo**

USA

**Robert Craigie**

USA

**Jie Cui**

Singapore

**Nathan Cummins**

USA

**Atze Das**

Netherlands

**Siddhartha Datta**

USA

**Matthew Daugherty**

USA

**Rob De Boer**

Netherlands

**Ward De Spiegelaere**

Belgium

**Zeger Debyser**

Belgium

**Gregory Del Prete**

USA

**Cynthia Derdeyn**

USA

**Benjamin Descours**

France

**Felipe Diaz-Griffero**

USA

**Lucy Dorrell**

UK

**Joseph Dougherty**

USA

**Maryam Ehteshami**

USA

**Simon Elsasser**

UK

**Auda Eltahla**

Australia

**Stephane Emiliani**

France

**Alan Engelman**

USA

**Le Rouzic Erwann**

France

**Joakim Esbjornsson**

Sweden

**Jose Este**

Spain

**Hung Fan**

USA

**Ariberto Fassati**

UK

**David Favre**

USA

**Boris Fehse**

Germany

**Guido Ferrari**

USA

**Cadhla Firth**

Australia

**Donald Forthal**

USA

**Eric Freed**

USA

**Simon Frost**

UK

**Axel Fun**

UK

**Feng Gao**

USA

**Victor J. Garcia**

USA

**Anne Gatignol**

Canada

**Silvana Gaudieri**

Australia

**Sandra Gessani**

Italy

**Robert Gifford**

UK

**Nicolas Gillet**

Belgium

**Thomas Gilmore**

USA

**Stephen Goff**

USA

**Maureen Goodenow**

USA

**Heinrich Gottlinger**

USA

**Thomas Gramberg**

Germany

**Lachlan Gray**

Australia

**Patrick Green**

USA

**Harriet Groom**

UK

**Ravindra Kumar Gupta**

UK

**Phalguni Gupta**

USA

**Hillel Haim**

USA

**David Harrich**

Australia

**Reuben Harris**

USA

**Johnny He**

USA

**Georges Herbein**

France

**Elena Herrera Carrillo**

Netherlands

**Vanessa Hirsch**

USA

**Amnon Hizi**

Israel

**Daniel Hoffmann**

Germany

**J. Robert Hogg**

USA

**Edward Hollox**

UK

**Margaret Hosie**

UK

**Stephen Hughes**

USA

**Eric Hunter**

USA

**James Hurley**

USA

**Fumitoshi Ishino**

Japan

**Yasumasa Iwatani**

Japan

**Martin Roelsgaard Jakobsen**

Denmark

**Shahid Jameel**

India

**Richard Jenner**

UK

**Dong-Yan Jin**

Hong Kong

**Welkin Johnson**

USA

**Stefan Jonsson**

Iceland

**Albert Jordan**

Spain

**Luis Kanzaki**

Brazil

**Jonathan Karn**

USA

**Fatah Kashanchi**

USA

**George Kassiotis**

UK

**Michael Katze**

USA

**Haig Kazazian**

USA

**Sarah Keane**

USA

**Anthony Kelleher**

Australia

**Julia Kenyon**

UK

**Matthew Kesic**

USA

**Yuri Khudyakov**

USA

**Rosemary Kiernan**

France

**Baek Kim**

USA

**Frank Kirchhoff**

Germany

**Andrea Kirmaier**

USA

**Zachary Klase**

USA

**P. J. Klasse**

USA

**Linda Klavinskis**

UK

**Simon Kollnberger**

UK

**Dusan Kordis**

Slovenia

**Christian Korner**

USA

**Yoshio Koyanagi**

Japan

**Christine Kozak**

USA

**Anil Kumar**

USA

**Gopal Kundu**

India

**Mamuka Kvaratskhelia**

USA

**Bernard Lafont**

USA

**Nathaniel Landau**

USA

**Mani Larijani**

Canada

**Marie Larsson**

Sweden

**Marc Lavigne**

France

**Vicki Lawson**

Australia

**Christine Leib-Mösch**

Germany

**Jonathan Leis**

USA

**Thomas Leitner**

USA

**Sharon Lewin**

Australia

**George Lewis**

USA

**Chen Liang**

Canada

**Jeffrey Lifson**

USA

**Maxine Linial**

USA

**Shan-Lu Liu**

USA

**Jeremy Luban**

USA

**Paul Luciw**

USA

**Marina Lusic**

Germany

**Rebecca Lynch**

USA

**Dixie Mager**

Canada

**Gkikas Magiorkinis**

UK

**Renaud Mahieux**

France

**Johnson Mak**

Australia

**Simon Mallal**

USA

**Fabrizio Mammano**

France

**Alessandro Marcello**

Italy

**Leonid Margolis**

USA

**Florence Margottin-Goguet**

France

**Enrique Martin-Gayo**

USA

**Cynthia Masison**

USA

**Masao Matsuoka**

Japan

**Patrick Matthias**

Switzerland

**Jens Mayer**

USA

**Aine Mcknight**

UK

**Paul Mclaren**

Canada

**Jean-Michel Mesnard**

France

**Thibault Mesplede**

Canada

**Henri Alexander Michaud**

France

**Takayuki Miyazawa**

Japan

**Hoi Ping Mok**

UK

**M. Anthony Moody**

USA

**Penny Moore**

South Africa

**Arnaud Moris**

France

**Kazuhiro Morishita**

Japan

**Lynn Morris**

South Africa

**Walther Mothes**

USA

**Andrew J. Mouland**

Canada

**Barbara Mueller**

Germany

**Erik Mullers**

Sweden

**Jan Münch**

Germany

**Tsutomu Murakami**

Japan

**Emi Nakayama**

Japan

**Vivek Naranbhai**

UK

**Sonia Navas-Martin**

USA

**Thumbi Ndung’U**

UK

**Sergei Nekhai**

USA

**George Nelson**

USA

**Douglas Nixon**

USA

**Philip Norris**

USA

**Piotr Nowak**

Sweden

**Akihiko Okayama**

Japan

**Alex Olvera**

Spain

**Marcel Ooms**

USA

**Alexandra Oster**

USA

**Anand Pai**

USA

**Suresh Pallikkuth**

USA

**Massimo Palmarini**

UK

**Gianfranco Pancino**

France

**Maria Papathanasopoulos**

South Africa

**Vincent Parissi**

France

**Vinay Pathak**

USA

**Steven Patterson**

UK

**William Paxton**

Netherlands

**Brendan Payne**

UK

**Susan Payne**

USA

**Finn Skou Pedersen**

Denmark

**Isabela Pedroza-Pacheco**

UK

**Josué Pérez-Santiago**

USA

**Gaël Petitjean**

France

**Satish Pillai**

USA

**Vicente Planelles**

USA

**Stefan Pöhlmann**

Germany

**Guido Poli**

Italy

**Filippos Porichis**

USA

**Pantelis Poumbourios**

Australia

**Mike Powell**

USA

**Christopher Power**

Canada

**Vinayaka Prasad**

USA

**Otto Pritsch**

Uruguay

**Xuebin Qin**

USA

**Jan Rehwinkel**

UK

**William Reid**

USA

**Alan Rein**

USA

**David Rekosh**

USA

**Wolfgang Resch**

USA

**Andrew Rice**

USA

**Nadia Roan**

USA

**Marjorie Robert-Guroff**

USA

**Morgane Rolland**

USA

**Nicola Rose**

UK

**Itay Rousso**

Israel

**Ioulia Rouzina**

USA

**Helen Rowe**

UK

**Maria Rumlova**

Czech Republic

**Ivan Sadowski**

Canada

**Saveez Saffarian**

USA

**Manish Sagar**

USA

**Mineki Saito**

Japan

**Nitin Saksena**

Australia

**Jun-Ichi Sakuragi**

Japan

**Marco Salemi**

USA

**Rogier Sanders**

Netherlands

**Mario Santiago**

USA

**Quentin Sattentau**

UK

**Daniel Sauter**

Germany

**Sara Sawyer**

USA

**Michael Schindler**

Germany

**Timothy Schlub**

Australia

**Olivier Schwartz**

France

**Oliver Semmes**

USA

**George Shaw**

USA

**Ann Sheehy**

USA

**John Silke**

Australia

**Viviana Simon**

USA

**Marc Sitbon**

France

**Jordan Skittrall**

UK

**Jacek Skowronski**

USA

**Nicolas Sluis-Cremer**

USA

**Harold Smith**

USA

**Thomas Smithgall**

USA

**Redmond Smyth**

France

**Carolyn Stenbak**

USA

**Edward Stephens**

USA

**James Stivers**

USA

**Heribert Stoiber**

Austria

**Jonathan Stoye**

UK

**Klaus Strebel**

USA

**Justine Sunshine**

USA

**Dmitri Sviridov**

Australia

**Ronald Swanstrom**

USA

**William Switzer**

USA

**Gilda Tachedjian**

Australia

**Yasuhiro Takeuchi**

UK

**Rachael Tarlinton**

UK

**Harry Taylor**

USA

**Philip Tedbury**

USA

**Kok Keng Tee**

Malaysia

**Amalio Telenti**

USA

**Alice Telesnitsky**

USA

**Kenzo Tokunaga**

Japan

**Martin Tolstrup**

Denmark

**Bruce Torbett**

USA

**Greg Towers**

UK

**John Trowsdale**

UK

**Athe Tsibris**

USA

**Dorothy Tuan**

USA

**Klaus Uberla**

Germany

**David Van De Vijver**

Netherlands

**Thijs Van Montfort**

Netherlands

**Koen Van Rompay**

USA

**Jean-Pierre Vartanian**

France

**Vijayakumar Velu**

USA

**Bruno Verhasselt**

Belgium

**Jens Verheyen**

Germany

**Elisa Vicenzi**

Italy

**Michelle Vincendeau**

Germany

**Volker M. Vogt**

USA

**Max Von Kleist**

Germany

**Stephen Waggoner**

USA

**Abdul Waheed**

USA

**Mark Wainberg**

Canada

**Toshiki Watanabe**

Japan

**Sarah Welbourn**

USA

**Craig Wilen**

USA

**Lucas Willems**

Belgium

**Kenneth Witwer**

USA

**Li Wu**

USA

**Yoshihisa Yamano**

Jordan

**Shoji Yamaoka**

Japan

**Venkat Yedavalli**

USA

**Takeshi Yoshida**

Japan

**Alessia Zamborlini**

France

**John Zaunders**

Australia

**Steven Zeichner**

USA

**Yong-Hui Zheng**

USA

